# Grape Version 1: First prototype of the low-cost personal space weather station receiver

**DOI:** 10.1016/j.ohx.2022.e00289

**Published:** 2022-03-10

**Authors:** John Gibbons, Kristina Collins, David Kazdan, Nathaniel Frissell

**Affiliations:** a Case Western Reserve University, United States; b University of Scranton, United States

**Keywords:** Doppler, Citizen science, HamSCI, Low IF receiver, WWV

## Abstract

Crowd sourced data collection among the international community of amateur radio operators and shortwave listeners has great potential for addressing problems of under-sampling in the geospace system. Quantitative Doppler measurements of high frequency (HF) time standard stations, used in bottom side ionospheric sensing, have been accomplished using existing radio hardware belonging to volunteers in distributed campaigns. However, typical shortwave receivers cannot be put to ordinary use while these measurements are being taken, do not have standardized signal chains, and are generally too expensive to be purchased for the sole purpose of taking Doppler measurements. Here, we provide documentation for a low-cost intermediate frequency receiver, the Grape Version 1, which is designed specifically for measurements of North American time standard stations. Grape receivers can be easily constructed and deployed by amateur scientists in order to gain a deeper understanding of variations in radio propagation in their local environment. When compared over long periods and across distributed networks of stations, the resulting data yield insights on greater spatial and time scales. At the time of writing, several of these receivers have been deployed across the United States and are actively collecting data. These receivers form the first iteration of the Low-Cost Personal Space Weather Station network.

## Graphical abstract

**Figure:**
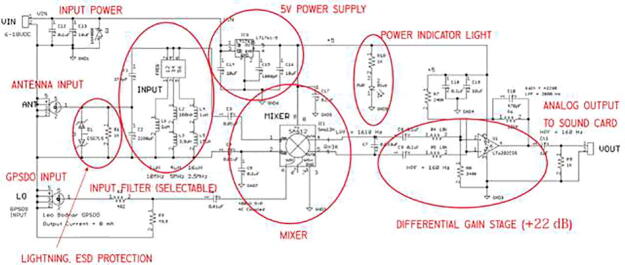

**Specifications table:**

**Table:** 

**Hardware name**	Grape V1/ Low Cost Personal Space Weather Station

**Subject area**	• Distributed Low-Cost Sensors for Geospace Environment • Educational Tools and Open Source Alternatives to Existing Infrastructure
**Hardware type**	• Field measurements and sensors • Electrical engineering and computer science
**Open source license**	TAPR Open Hardware License
**Cost of hardware**	Approximately $300 for full apparatus; $30 for populated mixer board
**Source file repository**	https://doi.org/10.17632/nbbhy2yxmz.1

## 1 Hardware in context

Fundamental questions in solar and space physics can be addressed through the development of new instrumentation and networks that can provide higher spatial and temporal resolution measurements of local, regional, and global scale geospace processes. This is the purpose of the Personal Space Weather Station (PSWS) network. A PSWS is a modular, multi-instrument, ground-based system that can be operated by and is affordable to individuals. Minimum required coverage is guaranteed through stations sited and managed by institutions, and augmented by citizen scientists in the amateur radio and shortwave listening communities [1].The PSWS network of instruments is easily expandable and can grow organically by engaging the general public.

**Fig. 1:**
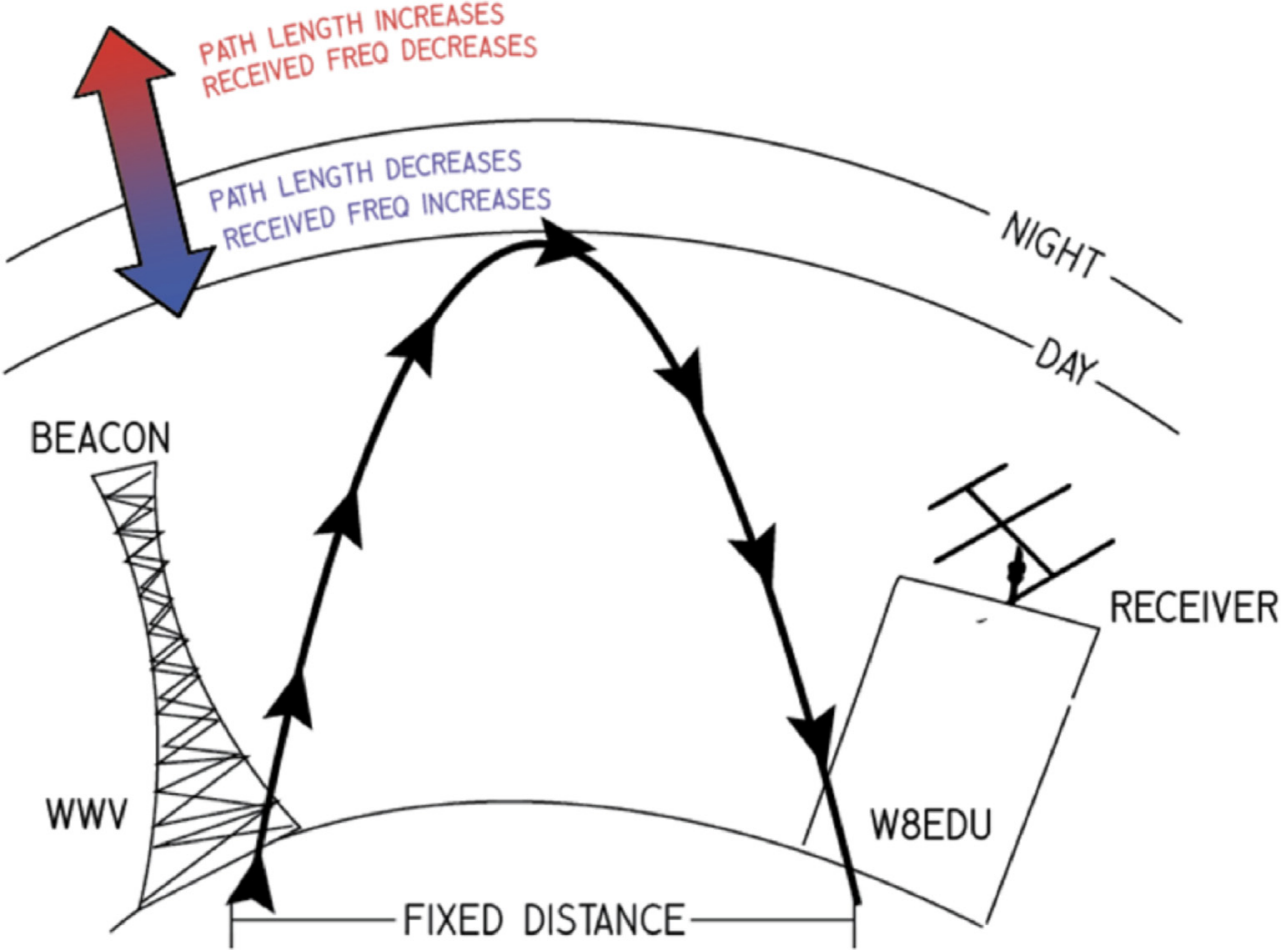
Simplified illustration of Doppler shift occurring during ionospheric skip propagation.

**Fig. 2:**
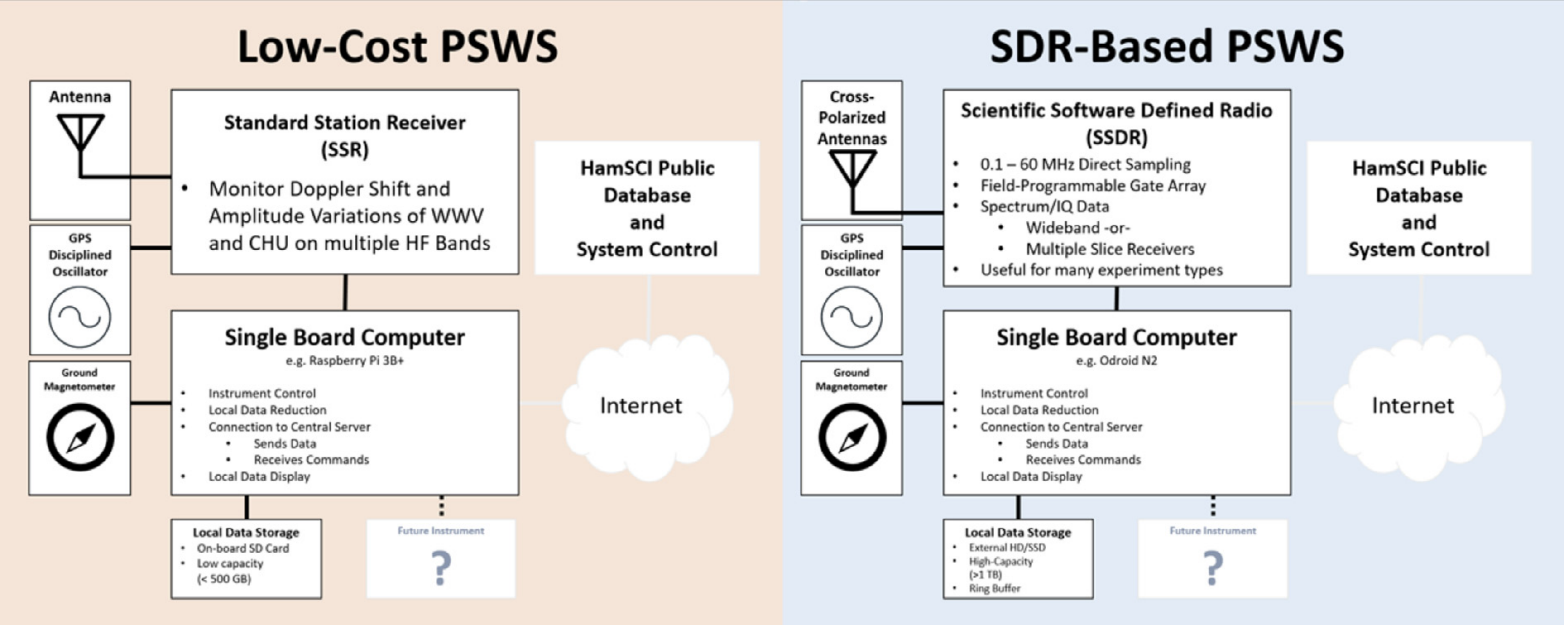
Two flavors of the Personal Space Weather Station. The Grape is on the left. The PSWS has a modular architecture: peripheral components, such as a ground magnetometer, can be added at a later date to enable additional geophysical data collection.

There are two variants of PSWS systems, as shown in Fig. 2: the narrowband Low-Cost PSWS, called the Grape Receiver, which is the subject of this paper; and the wideband SDR-Based PSWS, called the TangerineSDR. The Grape’s task is to enable Doppler measurements of time standard stations. These are stations run by governmental and laboratory organizations which transmit coded and audio time announcements. In the United States, stations WWV in Fort Collins, CO and WWVH in Kauai, HI transmit on 2.5, 5, 10 and 15 MHz. WWV also adds 20 and 25 MHz. In Canada, CHU transmits on 3.33, 7.85, and 14.67 MHz. These stations typically transmit extremely precise carrier frequencies, which allow them to be used as beacon stations for remote sensing. This carrier frequency is shifted when it reaches a receiving station according to the path it takes through the ionosphere. Fig. 1 shows a simplified model, assuming a single hop propagation mode. As the virtual height at the point of reflection rises, the path length increases; the wavefronts of the carrier are stretched farther apart and the received frequency drops. When the virtual height falls, the path length decreases and the received frequency increases. By comparing the received frequency to a precise local oscillator, such as a GPS-disciplined oscillator (GPSDO), a proxy measurement of the shift in ionospheric height may be obtained.

The technique of HF Doppler measurements has been employed for many decades. An important application of this measurement is the study of traveling ionospheric disturbances (TIDs). TIDs are quasi-periodic variations in ionospheric F-region electron density, and are generally classified as either Medium Scale TIDs (MSTIDs, horizontal velocities between 100 to 250 m ${\text{s}}^{-1}$, periods between 15 min to 1 h, and horizontal wavelengths of several hundred km) or Large Scale TIDs (LSTIDs, horizontal velocities between 400 to 1000 m ${\text{s}}^{-1}$, periods between 30 min to 3 h, horizontal wavelengths greater than 1000 km) [2], [3], [4]. Understanding the characteristics of TIDs can provide insight into the transport of energy between different regions of the atmosphere and the ionosphere [5], and currently TID modeling efforts such as [6] need to be validated with actual measurements. State-of-the-art TID detection systems are generally capable of detecting LSTIDs but may lack the density of instruments necessary to detect MSTIDs. The system described in [7], which detects TIDs in real time by means of vertical ionospheric measurements, is unable to reliably detect MSTIDs without a denser network of instruments (separated by less than typical wavelengths of MSTIDs) and cadences of measurements shorter than 5 min. The detection of MSTIDs with the system described in this paper is a target of current research. Improved TID detection will, in turn, support the exploration of frontier questions in atmosphere–ionosphere coupling by helping to establish the relationships between TIDs and acoustic gravity waves (AGWs).

Today, with the advantage of cheaper instrumentation and computational power, synchronized HF Doppler measurements can be conducted by many stations simultaneously. This advance is due not only to the availability of inexpensive single-board computers such as the Raspberry Pi, but also to the advent of inexpensive GPS-disciplined oscillators. Taken together, fleets of individual stations can be considered as a single meta-instrument (i.e., an instrument made of contributions from many individual instruments), capable of observing traveling ionospheric disturbances and other ionospheric perturbations on a continental or global scale.

The first light of the citizen science meta-instrument was a pilot experiment in October 2019, the Festival of Frequency Measurement, held to celebrate WWV’s centennial. In this one-day campaign, participants across the United States used their amateur radio equipment to gather frequency estimation data, as described in [8]. While that study demonstrated a high correlation between nearby stations and showed promising results despite the variety of experimental apparatus used, it also showed its limits: the volunteers’ equipment could not be used for ordinary activity during the experiment, which would be prohibitive for long-term data collection; the varied signal chains resulted in errors which required some data-sets to be culled; and the need for shortwave receivers costing hundreds or thousands of dollars created a barrier to entry that restricted the scope of the data collection to members of the amateur radio and shortwave listening communities, despite the fact that most features of these receivers are surplus to our needs for this particular experiment.

To address these limitations, we present the Grape Version 1, a low-cost, purpose-built low IF receiver for Doppler observations on 2.5, 5 and 10 MHz. The Grape Version 1 will be succeeded by the Grape Version 2, which will include additional channels for simultaneous monitoring of other frequencies of WWV (15 MHz) and CHU (3.33 MHz, 7.85 MHz and 14.67 MHz).

*Nota bene:* Where possible, amateur radio callsigns are used herein, in addition to names, in order to specify individuals and club stations. Because these callsigns are unique and persistent identifiers, they support the Findability criterion of FAIR Data principles. The authors’ callsigns are N8OBJ, KD8OXT, AD8Y, and W2NAF, respectively.

## 2 Hardware description

**Fig. 3:**
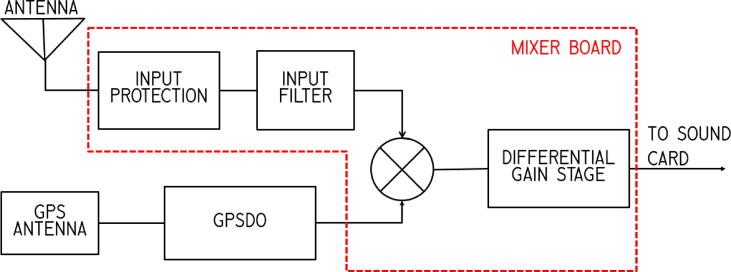
Architecture block diagram showing the signal chain of the Grape receiver. The power connections are not shown.

The Grape is a very simple heterodyne receiver, as illustrated in Fig. 3. The GPSDO signal acts as the local oscillator, whose signal, set to 1 kHz below the carrier frequency of interest, is mixed with the filtered input signal. The output of this operation is an intermediate frequency at the difference frequency of 1.000 kHz. This is the signal that is used to measure the Doppler shift of the carrier frequency.

In the use case it is designed for, its primary advantage is low cost. Most of the system cost derives from the GPSDO and the Raspberry Pi, rather than the mixer board. The cost of the mixer board itself is about $30, and it stands in for a transceiver which may cost hundreds or thousands of dollars, e.g., the Icom 7610. There are few communications receivers which provide for high-accuracy frequency references such as a GPSDO, and the ones that do are expensive. Additionally, the Grape’s signal chain is simple and easily characterized, as opposed to the “black box” posed by the more convoluted signal chain of a conventional transceiver. The Grape is an analog receiver and will have no more time delay incurred than that from its filters. Most contemporary communications receivers use digital filtering with phase and group delays that distort measurements for the Grape’s purposes.

There are additional aspects of the Grape which make it more suitable for these measurements than most commercial receivers. Very few communications receivers have provisions for shutting off automatic gain control (AGC), which makes absolute received signal level measurements impossible. The Grape has no AGC; at risk of overload, it can act as a field strength measuring device. Frequency accuracy is a function of the GPSDO rather than of the Grape. Grape selectivity will not be expected to be as high as that of an excellent communications receiver, but there are few adjacent-channel interfering signals on the frequencies of interest in this use case.

The Grape is designed to monitor 2.5, 5, or 10 MHz, but could be customized to another frequency in the HF band by modifying the inductance and/or capacitance values of the input filter. In use cases where the precision of a GPSDO is not required, an inexpensive oscillator circuit can be substituted for the local oscillator. Such uses include relative time of flight measurements on the transmitter’s modulations such as the second ticks. Purpose-designed modulations such as those from the WWV/H Scientific Modulation Working Group [9] also fall into this category. It can also be used as the basis for a simple classroom demonstration of a heterodyning receiver.

The open-source software accompanying the Grape generates daily records of received signal amplitude and frequency shift, in the form of both plots and.CSV files.

**Fig. 4:**
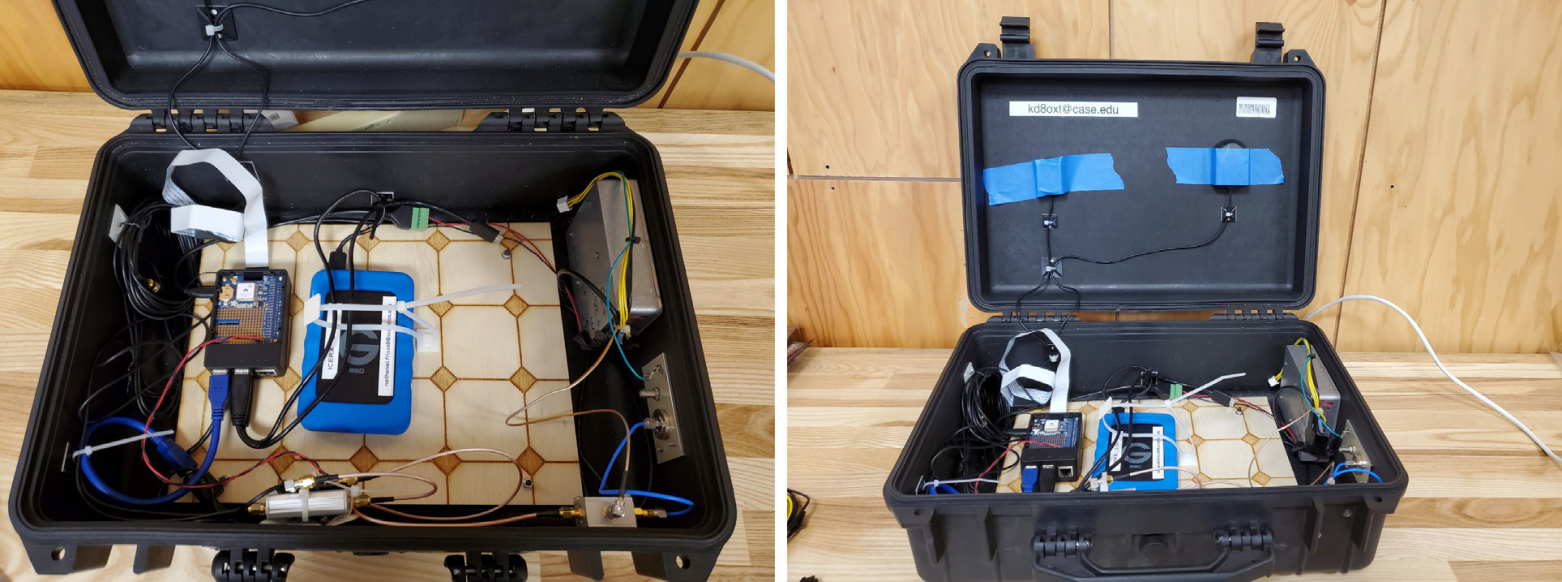
Grape V1 station in the process of being packaged in a ruggedized box. This installation will use a DX Engineering RF-PRO-1B®active magnetic loop antenna. The system’s position is recorded using an Adafruit Ultimate GPS HAT. Image credit: Ben Kaufman KB3VSC.
• **Carrier dropout monitoring.** In addition to checking for the absence of a carrier, the Grape could be used to check for the outage of a carrier in a particular sequence as a form of communication, as was used in the CONELRAD system in the 1950s.
• **Educational applications.** The Grape is a good introductory surface-mount soldering kit, appropriate to high school or college level, and can be deployed as a long-term classroom experiment to stimulate interest in space weather.

Inside and outside of the original user community, some uses for the Grape V1 include:

• **Constant monitoring on single frequency, with modifications to the board as described above.** Alerts can be set if the amplitude or frequency of the signal goes outside an expected range. In the amateur radio space, it could be modified to monitor the band edge, and indicate transmissions outside allocated spectrum.
• **Propagation studies.** The Grape is small and highly portable, and could be used to check for presence of a beacon signal on a particular frequency at a given location, provided that antennas are also consistent. An example of a Grape packaged for portability is shown in Fig. 4.

## 3 Design files

### 3.1 Design files summary

**Fig. 5:**
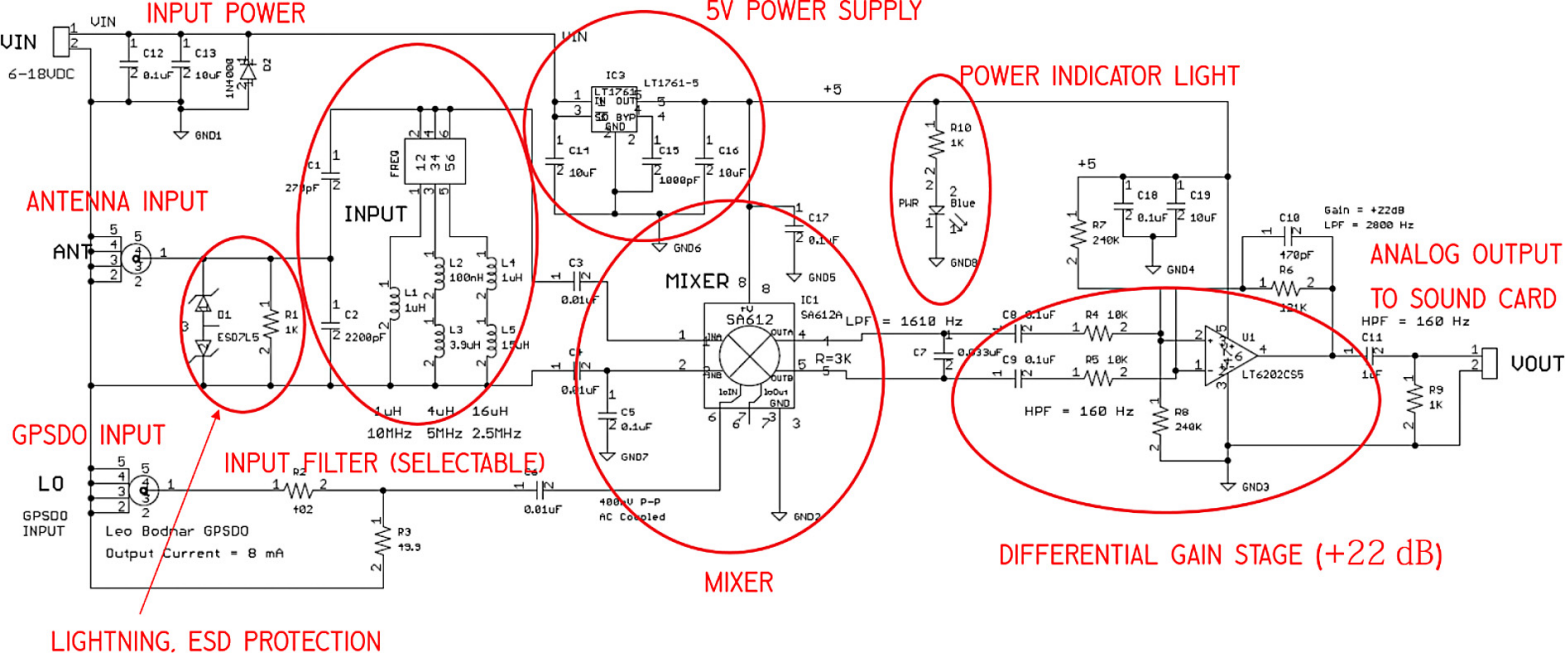
Annotated Grape 1.0 schematic.

The files are summarized in Table 1. The root directory includes the OS image for the Raspberry Pi (Grape1_OS.Gen1.img-002.zip) and information on the filename conventions for PSWS nodes (PSWS_FileNameRequirementsV0_15.pdf and PSWS_Node_Info.jpg). The main folder, Grape_Gen1_PSWS, includes a modified version of fldigi (FLDigi-4.1.13), a folder of python scripts for node setup (PSWSsetup), and installation instructions (MakeGrape1OSImageV1.01.pdf), as well as supplemental files. Design and manufacturing files, including photographs, bills of materials and Gerber files, are found in the subfolder WWV_Radio_V1.11 (See Fig. 5).

**Table 1:** Design files.

**File**			**Description**	**Open Source License**	**File Location**
Grape1_OS_Gen1.img-002.zip			OS Image		
PSWS_FileNameRequirementsV0_15.pdf			Naming conventions, file structures, descriptions		
PSWS_Node_Info.jpg			Example node data		
	FLDigi-4.1.13		Modified version of fldigi		
	Grape1_OS_Dist2a.txt		Hash codes for OS		
	Grape1FreqChange.pdf		Instructions to switch beacon frequency		
	GrapeGen1_SuppliesList.pdf		Overall Bill of Materials		
	MakeGrape1OSImageV1_01.pdf		Instructions to install OS image		
	OS_Image		Instructional screenshots for OS installation		
	PSWS_GrapeLowCostBlockDiagram.pdf		Block diagram		
	PSWSsetup		Folder of python scripts to set up and run node		
	StationUsage.pdf		Instructions to pause station operation		
		DigiKey_Bom_8574962.csv	Electronics BOM with Digikey part numbers		
		eagle.epf	EAGLE project file		
		Gerbers V1.11	Gerber files		
		GrapeGen1Pics	Construction photos of soldered board		
		WWV_Grape1_BOM.txt	Part list organized by component type		
		WWV_Radio_V1_11.bom	EAGLE bill of materials		
		WWV_Radio_V1_11.brd	EAGLE board file		
		WWV_Radio_V1_11.sch	EAGLE schematic file		
		WWV_Radio_V1_11_parts.txt	Electronics BOM in text format		
Grape_Gen1_PSWS	WWV_Radio_V1_11	WWV_Radio_V1_11_Sch.pdf	Schematic diagram	TAPR OHL	10.17632/nbbhy2yxmz.1

## 4 Bill of materials

### 4.1 Project components

In addition to the mixer board, a functional PSWS requires a tunable GPS-disciplined oscillator, a central computer, and an HF antenna. A monitor, mouse and keyboard will also be required to interface with the Raspberry Pi. The GPSDO can be programmed from an external computer.

### 4.2 Electronic components

The total cost of electronic components for the board is $22.49, as listed in Table 3.

**Table 3:** Electronic components.

**Manufacturer Part Number**	**Manufacturer**	**Digi-Key Part Number**	**Qty**	**Unit Price**	**Total**	**Reference Designator**	**Description**
C0805C104M3RACTU	KEMET	399-8000-1-ND	6	0.1	$0.60	C5, C8, C9, C12, C17, C18	CAP CER 0.1UF 25 V X7R 0805
C0805C103J5RAC7210	KEMET	399-17616-1-ND	3	0.1	$0.30	C3, C4, C6	CAP CER SMD 0805.01UF X7R 5% 50
885012207095	Würth Elektronik	732-8077-1-ND	1	0.1	$0.10	C7	CAP CER 0.033UF 50 V X7R 0805
885012207103	Würth Elektronik	732-7678-1-ND	1	0.27	$0.27	C11	CAP CER 1UF 50 V X7R 0805
C2012X5R1E106M125AB	TDK Corporation	445-5985-1-ND	4	0.33	$1.32	C13, C14, C16, C19	CAP CER 10UF 25 V X5R 0805
08055C271KAT2A	AVX Corporation	478-1364-1-ND	1	0.22	$0.22	C1	CAP CER 270PF 50 V X7R 0805
885012207084	Würth Elektronik	732-8066-1-ND	1	0.1	$0.10	C10	CAP CER 470PF 50 V X7R 0805
885012207086	Würth Elektronik	732-8068-1-ND	1	0.1	$0.10	C15	CAP CER 1000PF 50 V X7R 0805
885012207088	Würth Elektronik	732-8070-1-ND	1	0.1	$0.10	C2	CAP CER 2200PF 50 V X7R 0805
ERJ-6ENF49R9V	Panasonic	P49.9CCT-ND	1	0.1	$0.10	R3	RES SMD 49.9 OHM 1% 1/8 W 0805
ERJ-6ENF4020V	Panasonic	P402CCT-ND	1	0.1	$0.10	R2	RES SMD 402 OHM 1% 1/8 W 0805
ERJ-6ENF1001V	Panasonic	P1.00KCCT-ND	3	0.1	$0.30	R1, R9, R10	RES SMD 1 K OHM 1% 1/8 W 0805
RC0805JR-0710KL	Yageo	311-10KARCT-ND	2	0.1	$0.20	R4, R5	RES SMD 10 K OHM 5% 1/8 W 0805
CRCW0805121KFKEA	Vishay Dale	541-121KCCT-ND	1	0.1	$0.10	R6	RES SMD 121 K OHM 1% 1/8 W 0805
CRCW0805240KFKEA	Vishay Dale	541-240KCCT-ND	2	0.1	$0.20	R7, R8	RES SMD 240 K OHM 1% 1/8 W 0805
1N4001RLG	ON Semiconductor	1N4001RLGOSCT-ND	1	0.2	$0.20	D2	DIODE GEN PURP 50 V 1A DO41
ESD7L5.0DT5G	ON Semiconductor	ESD7L5.0DT5GOSCT-ND	1	0.72	$0.72	D1	TVS DIODE 5 V 10.4 V SOT723
LQH31MN1R0K03L	Murata Electronics	490-6604-1-ND	2	0.71	$1.42	L1, L4	FIXED IND 1UH 175MA 637 MOHM SMD
LQH31MN3R9K03L	Murata Electronics	490-16851-1-ND	1	0.68	$0.68	L3	FIXED IND 3.9UH 125MA 1.95 OHM
LQH31MN150J03L	Murata Electronics	490-11672-1-ND	1	0.71	$0.71	L5	FIXED IND 15UH 90MA 3.9 OHM SMD
LQW31HNR10J03L	Murata Electronics	490-12091-1-ND	1	0.65	$0.65	L2	FIXED IND 100NH 230MA 300 MOHM
APHCM2012VBC/D	Kingbright	754-2118-1-ND	1	0.54	$0.54	PWR	LED BLUE CLEAR 2SMD
LT1761IS5-5#TRMPBF	Analog Devices Inc.	LT1761IS5-5#TRMPBFCT-ND	1	2.95	$2.95	IC3	IC REG LINEAR 5 V 100MA TSOT23-5
LT6202CS5#TRMPBF	Analog Devices Inc.	LT6202CS5#TRMPBFCT-ND	1	4.19	$4.19	U1	IC OPAMP GP 1 CIRCUIT TSOT23-5
SA612AD/01,118	NXP USA Inc.	568-5045-1-ND	1	2.49	$2.49	IC1	IC MIXER 500MHZ UP CONVRT 8SO
CON-SMA-EDGE-S	RF Solutions	CON-SMA-EDGE-S-ND	2	1.69	$3.38	ANT, LO	CONN SMA JACK STR EDGE MNT
67997-206HLF	Amphenol ICC (FCI)	609-3234-ND	1	0.35	$0.35	FREQ	CONN HEADER VERT 6POS 2.54MM
QPC02SXGN-RC	Sullins Connector Sol’ns	S9337-ND	1	0.1	$0.10	JMP1	CONN JUMPER SHORTING.100” GOLD

## 5 Build instructions

### 5.1 Mixer board assembly

**Fig. 6:**
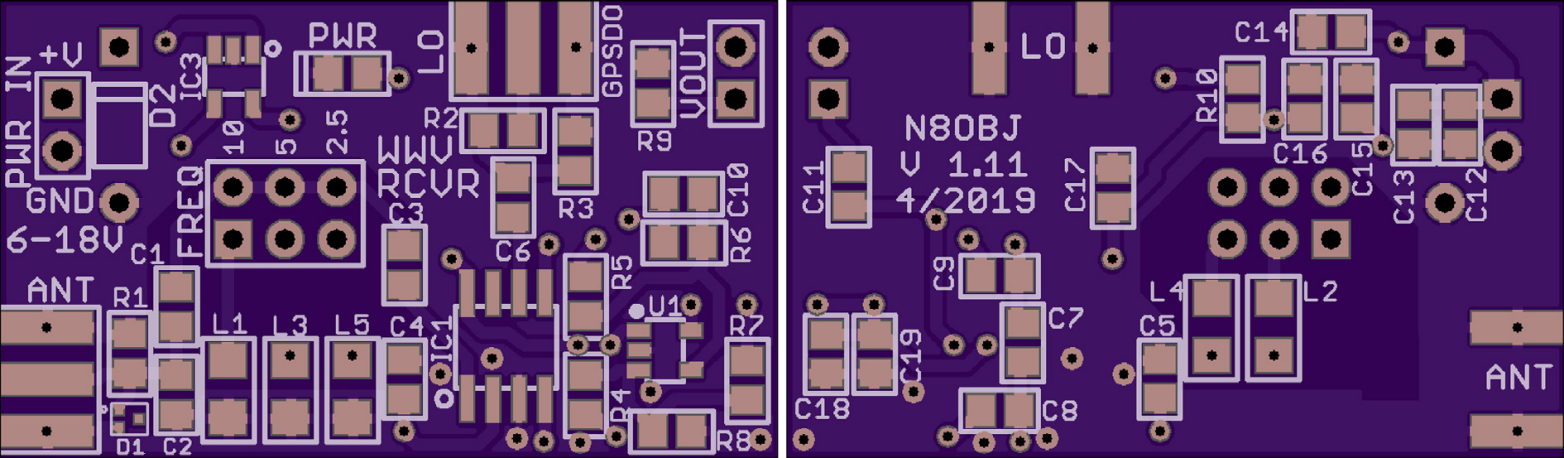
Front and back of Grape 1 PCB.

**Fig. 7:**
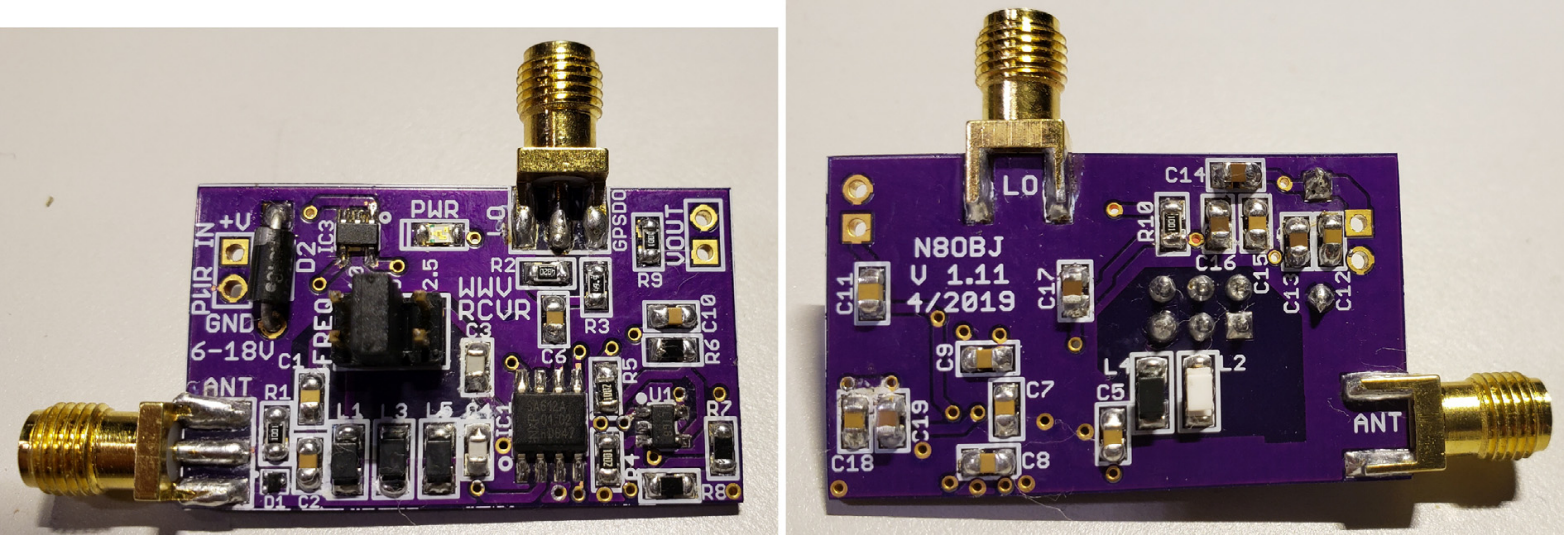
Populated circuit board.

The unpopulated circuit board may be purchased from OSHPark, as noted in Table 2, and populated with the parts listed in Table 3. The printed circuit board is shown in Fig. 6, and the populated board is shown in Fig. 7.

**Table 2:** Bill of Materials, not including antenna.

**Designator**	**Component**	**Number**	**Source**	**Unit Cost**	**Total Cost**
Grape V1	Mixer board	1	OSHPark	$6.60	$6.60
	Electronic Components (See below)		Digikey		$22.49
Raspberry Pi 4B	Raspberry Pi 4B with 4 GB memory	1	Amazon	$68.49	$68.49
Samsung MB-MJ64GA/AM	High endurance 64 GB micro SD card	1	Amazon	$14.99	$14.99
Smraza B07W5VQYG9	Case and cables for Raspberry Pi	1	Amazon	$19.99	$19.99
Sabrent AU-MMSA	USB sound adapter	1	Amazon	$7.98	$7.98
Leo Bodnar Mini	Programmable GPSDO	1	Leo Bodnar	$140.37	$140.37
DZS Elec RG316	4- SMA-M to SMA-M cable	1	Amazon	$7.49	$7.49
B00COW5E3A	4- SMA-M to SO-239 cable	1	Amazon	$6.80	$6.80
Belkin AUX	1/8- Stereo cable (10–12 inches)	1	Amazon	$8.99	$8.99
				**Total Cost:**	$**304.19**

A few recommendations for hand assembly: First, the reader is advised to purchase extra components in case of loss or damage. Assembling the board in a tray or cookie sheet with low sides may be helpful for keeping track of parts. One should use a soldering iron with a small tip (e.g., the Hakko FX888D-23BY Digital Soldering Station with the T18-BR02 tip) and keep the tip clean. When soldering surface mount parts, it is easiest to put solder on one pad then mount the part, soldering that pad first in order to adhere the part to the board, then soldering other pins. Hold each part gently with tweezers when placing it on the board. Using a small diameter spool of solder makes assembly easier as well.

The orientation of the mixer IC (U1) is indicated by a small white circle on the PCB near pin 1. The package will only identify the orientation by a slight slant on one edge of the package, which indicates which side pin 1 is on. It should be oriented with the slant toward the outside of the PCB. Looking from the top of the IC with the slant on the left side, pin 1 is on the top left and numbers progress counter-clockwise around the package (like any standard DIP IC package).

### 5.2 Mixer board testing

Once the board is assembled, it can be bench tested with a Leo Bodnar GPSDO, RF signal generator, power supply and oscilloscope. First, apply 6–18 V DC to the VIN power pins and verify that the power indicator LED turns on. At this point the board should typically draw about 7.2 mA of current. If the LED lights, check the linear regulator’s (IC3 pin 5) output voltage – it should be between 4.9 V and 5.2 V. Be careful not to short out the regulator and damage it.

If all that checks out, it is time to do a functional test. To do this, connect the Leo Bodnar GPSDO to the LO input on the board and set the frequency to 9.999 MHz with an output drive current of 8 mA.

Set the J1 JMPR on the Grape PCB to the 10 position and connect a RF signal generator to the ANT input jack. Set the output frequency to 10.000 MHz and the output level to 100 μV_peak_ (not RMS). Next, connect the oscilloscope to the audio output VOUT (pin 1 is the signal output, pin 2 is ground). On the oscilloscope, you should observe a 1 kHz sine wave with an amplitude of approximately 75–100 mV (depending on the skew of the LC part values in the front end filter). If you don’t have access to an oscilloscope, headphones can be used to verify the presence of a 1 kHz tone.

### 5.3 System assembly

**Fig. 8:**
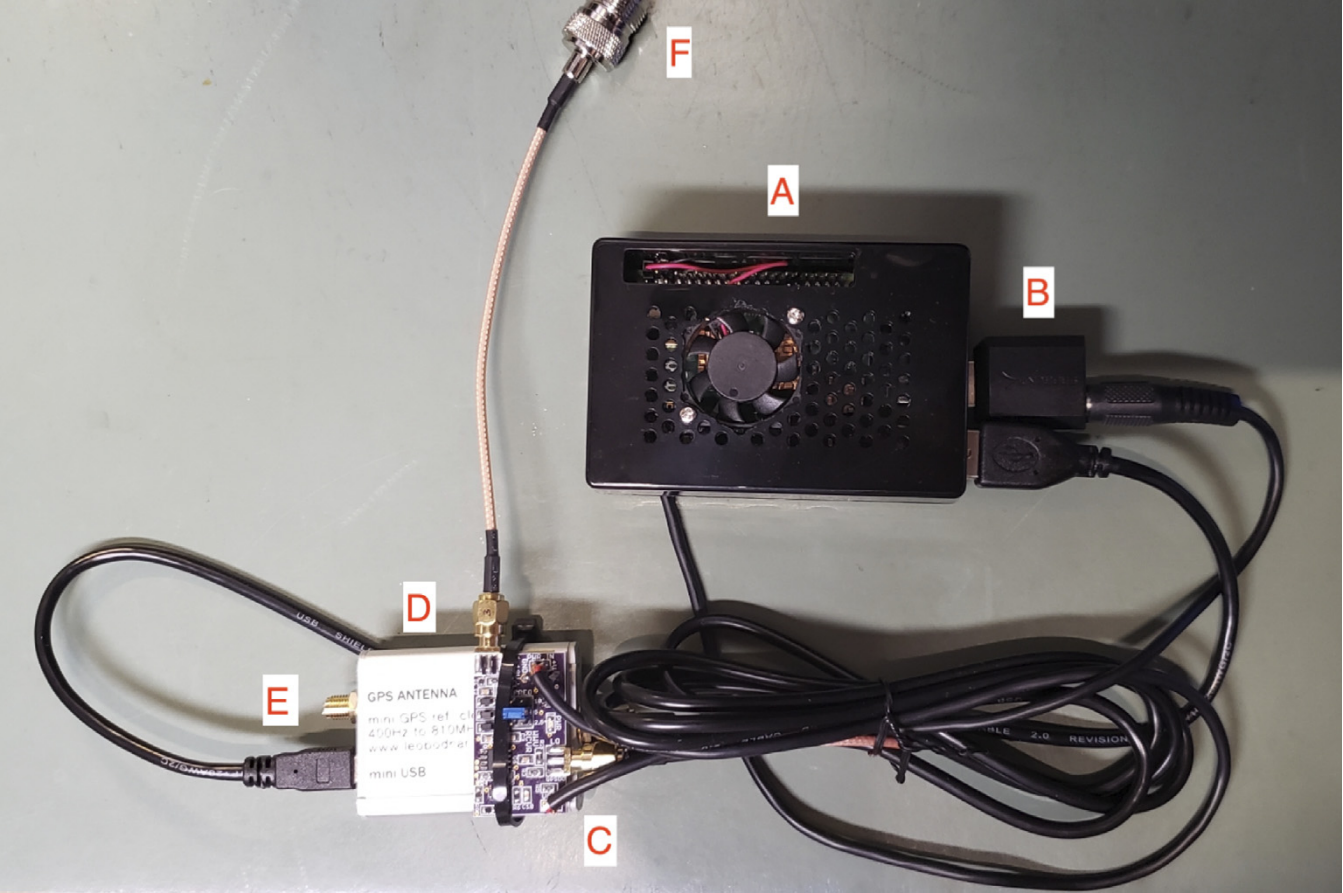
Built system, power connections and antennas not shown. (A) Raspberry Pi 4B with case and fan; (B) USB sound card interface; (C) Grape V1 board; (D) Leo Bodnar GPSDO; (E) SMA connector for GPS antenna; (F) SO-239 connection to receive antenna.

**Fig. 9:**
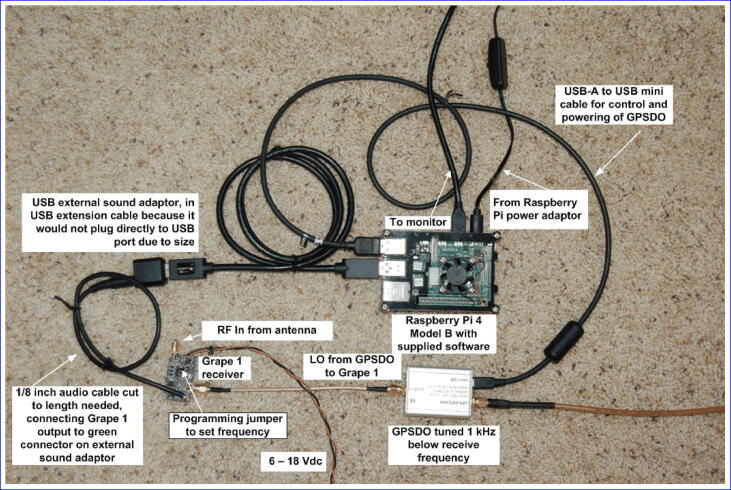
Built system with additional accessories and external power to Grape board. Image credit: Jim Farmer K4BSE.
• The GPSDO is powered from the Raspberry Pi using the included USB cable. (D) Plug the GPSDO USB cable into the lower left USB connector on the Raspberry Pi (diagonally across from the audio USB adapter).
• The GPS antenna (included with the Bodnar) is connected to the GPSDO. The antenna itself is not shown in Fig. 8. (E)
• The antenna input on the Raspberry Pi is connected to an appropriate HF antenna using an SMA – SO239 cable (F). The receive antenna (assumed to use a PL-259 connector) is further discussed in Section 5.4.

Once the mixer board has been assembled and tested, the system should be assembled as shown in Fig. 8:

• The USB audio adapter is plugged into the Raspberry Pi (B). Facing the Raspberry Pi USB connectors, put it into the top right position. The audio cable is connected to the pink connector input of the USB audio adapter coming from the VOUT audio output of the mixer board.
• The power pins on the mixer board are wired to the 5 V pins on the Raspberry Pi. (C) Alternatively, the board can be powered externally (6–18 V DC) as shown in the construction in Fig. 9.

The Leo Bodnar GPSDO is powered from the Raspberry Pi over USB, and draws 250 mA at 5 V. The mixer board draws 7.2 mA and can be wired to the 5 V pins on the Raspberry Pi (6–18 V DC is preferred, but 5 V will work). Thus, the primary 5 V power connection for the system is the power supply for the Raspberry Pi, and the total power draw is anywhere from 250 mA to 1.3 A, depending on what is running on the Raspberry Pi.

### 5.4 Receive antenna

Due to the variety of suitable HF antennas, detailed discussion of the receive antenna is outside the scope of this paper. We refer the reader to external resources, such as the *ARRL Antenna Book* [10]. Versatile HF antenna kits are also available. Alternatively, a long wire antenna may be used with a balun, such as a NooElec Balun One Nine, available on Amazon for $11.95. In this case, the SMA to SO-239 in Table 2 is replaced with a second SMA-M to SMA-M cable.

It is critical that the antenna be properly grounded to a real earth ground. For a wire antenna connected to one side of a balun, ensure that the other balun input is connected to earth ground. Poor grounding will deprive the system of a reference point, so the signal will be noisy and weak.

### 5.5 Software installation

The OS image for the Raspberry Pi is available on Mendeley with the design files. Burn this image to the micro SD card using balenaEtcher or a similar program, then insert the card into the Raspberry Pi and boot it. Once the Pi is running, follow the instructions provided with the OS image file to complete the installation. Detailed instructions are provided in MakeGrape1OSImage.pdf. This image includes a version of the open-source software fldigi 4.1.13 which we have modified to provide file labeling and metadata suitable for this project.

## 6 Operation instructions

**Fig. 10:**
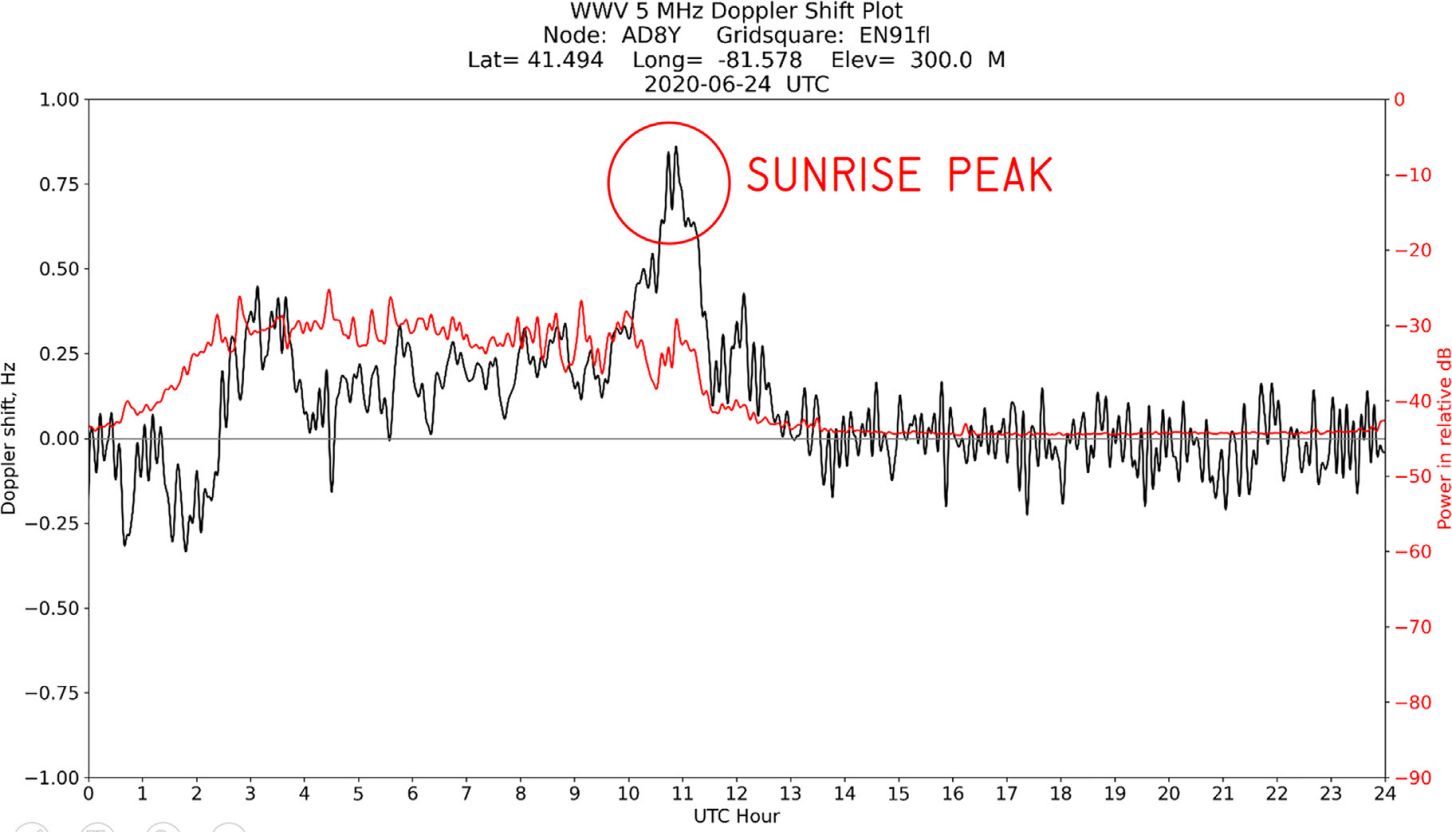
Example of daily plot, spanning 24 h from midnight to midnight UTC on 24 June 2020, with sunrise peak highlighted. The black line indicates the frequency shift of the carrier, and the red line indicates the amplitude of the signal. In Cleveland, where this data was taken, peaks indicative of traveling ionospheric disturbances (TIDs) are visible in the nighttime half of the plot, and the carrier signal becomes weak and noisy after sunrise. A plot like this is automatically generated by the Grape software each day. The data represented in this and other plots are archived at [11].

Once the software is installed and running, it will generate daily plots of the filtered signal in.PNG format and raw data in. CSV format. An example of one such plot is shown in Fig. 10.

Each day, when the operating system time hits 00:00:00 UTC, the customized version of FLDigi 4.1.13 begins running on this node to start a new day’s data collection file. This data file is stored in /PSWS/Srawdata/ as file analysisYYMMDD.csv, where YYMMDD is today’s new UTC date. The program then determines the beacon being monitored and checks and updates (if needed) the associated /PSWS/Sinfo/Beacon1 file (associated with the station Radio1) with the beacon identifier string (“WWV5”, for example). The program also reads system-related information (Node Number, Gridsquare, Latitude/ Longitude/Elevation, City State, RadioID, Beacon1) from this same /Sinfo directory. This is done to create the first line header info to aid in the plotting of this data file in the future (initially next day). If the frequency being monitored doesn’t match any of the 9 known beacons, it declares it as an ‘unknown’ beacon and is saved/plotted accordingly (with filename support indicating this).

Data collection continues in this file for the entire UTC day. If the data collection process is interrupted or stopped, the data is saved. When the process restarts, the new data gets appended to this same analysisYYMMDD.csv file where the data collection was interrupted as long as the UTC date is still the same. This allows for system down time, maintenance, system crashes, etc. The beacon frequency data is created and stored at a one second cadence to the.csv file by the data collection program. This gives 86,400 data entries per day in this file.

To pause data collection during the course of the day—for example, if one wishes to use the antenna for radio operation—the fldigi program may be set to NULL mode and the antenna disconnected. Afterwards, reconnecting the antenna and setting fldigi to Freq Analysis mode will allow data collection to be seamlessly resumed.

Data collection continues throughout the day until the system clock hits 00:00:00 UTC again, and the process restarts.

Also at 00:00:00 UTC, a crontab job for the user pi kicks off a python program to check the data file for corruption and to rename, process and move a new version to the correct directory for submission to the central node server. If the frequency directory does not exist yet for the newly processed frequency file, it is created (with correct file permissions 664) and the data then stored there. If the beacon is not in the list of known beacons, it’s named ‘unknown’ and stored as such. The script also removes the Freq Err and dB(Vpk) data columns from the file to be submitted to the server as well (these numbers can be derived from the data already there and are therefore considered redundant). The original analysisYYMMDD.csv is left in the /Srawdata/ directory for future access and for plotting. If the filecheck program finds any problems, it fixes and stores them (in /Srawdata) and appends the .bad file extension to the original file. It is kept for future reference.

## 7 Validation and characterization

Once the system is running, some observation is required in order to verify that the resulting signals are geophysical in nature, rather than the result of local RF interference. As noted in Section 5.4, a proper connection to earth ground is imperative.

**Fig. 11:**
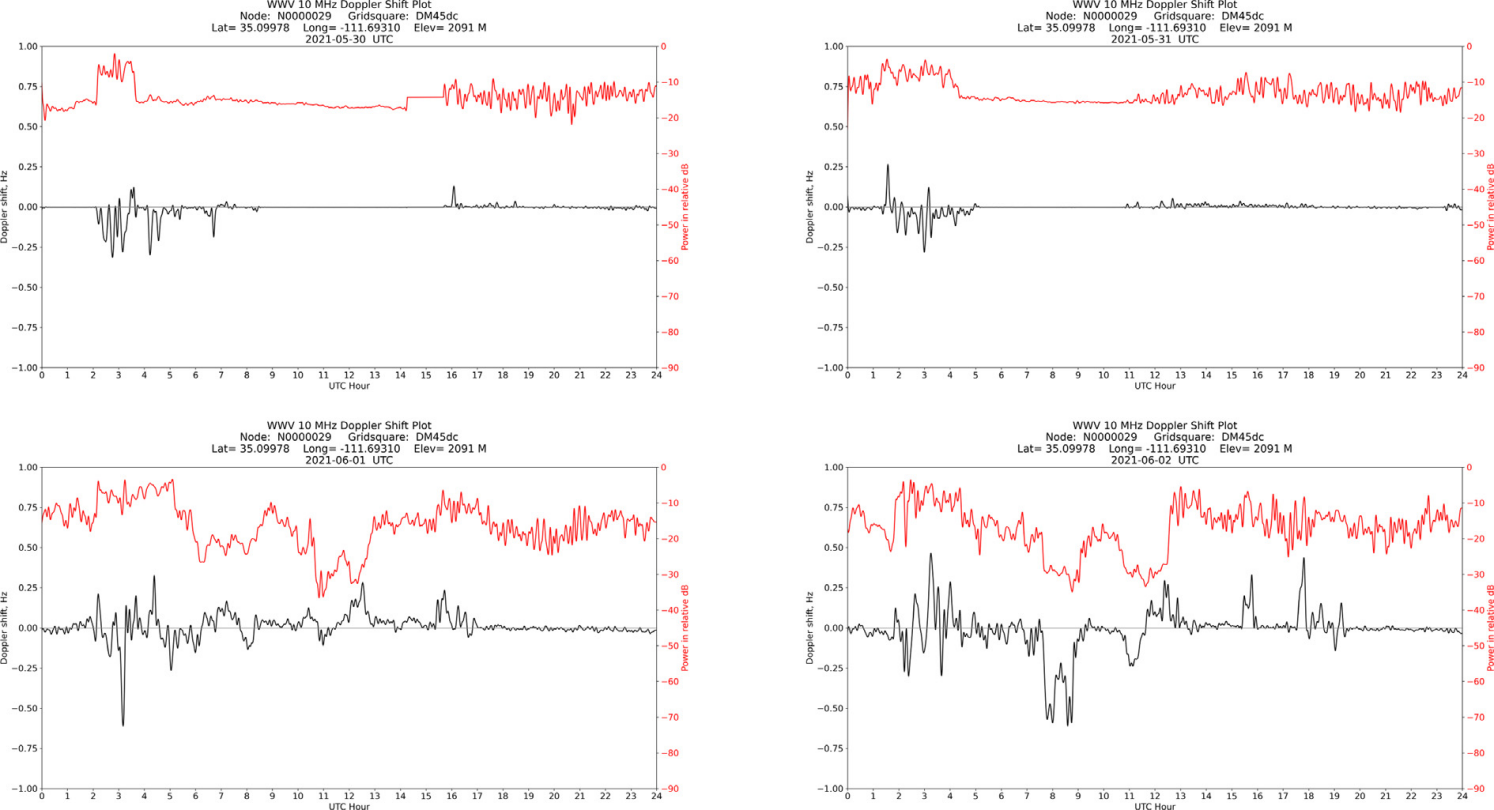
Four daily plots, recorded on consecutive days, from a station in Arizona. The first two show local interference from a 10 MHz receiver. Image credit: Joe Hobart W7LUX.

An example of local interference is shown in Fig. 11: the relatively flat red signal strength line in the top two plots show where leakage from a local standard and connected equipment is stronger than the signal from WWV. Even when the local signal is not stronger than WWV, a comparison of the top two plots with interference present and the bottom two interference free plots suggests a significant reduction in the measured amplitude of the Doppler shift received from WWV.

To reduce this interference, one can use standard techniques: better shielding, breaking ground loops, ferrite chokes, careful attention to balanced/unbalanced transformation, etc., or moving the antenna farther from the 10 MHz source. A transmission line common mode choke may also be used to minimize pickup of the local signal by the outer shield of the coax going to the WWV receiver. Double shielded coax between a 10 MHz standard and other equipment may be required.

One key indicator of a working system is the distinctive peak in the frequency plot shortly after local sunrise, when solar input to the ionosphere causes the virtual height to drop. This is a signature of the ionospheric diurnal variation. The ionosphere is formed primarily by photoionization of the neutral atmosphere due to solar ultraviolet (UV) energy. During the day, ionospheric electron densities increase due to photoionization; at night, ionospheric electron densities decrease due to recombination [12]. During the dawn transition, the increase in electron density causes a shortening of the propagation path with time, producing the positive Doppler sunrise peak signature observed between 10 and 12 UT on Fig. 10. As the ionosphere reaches daytime equilibrium, the Doppler shift returns to near-zero values for the rest of the day.

**Fig. 12:**
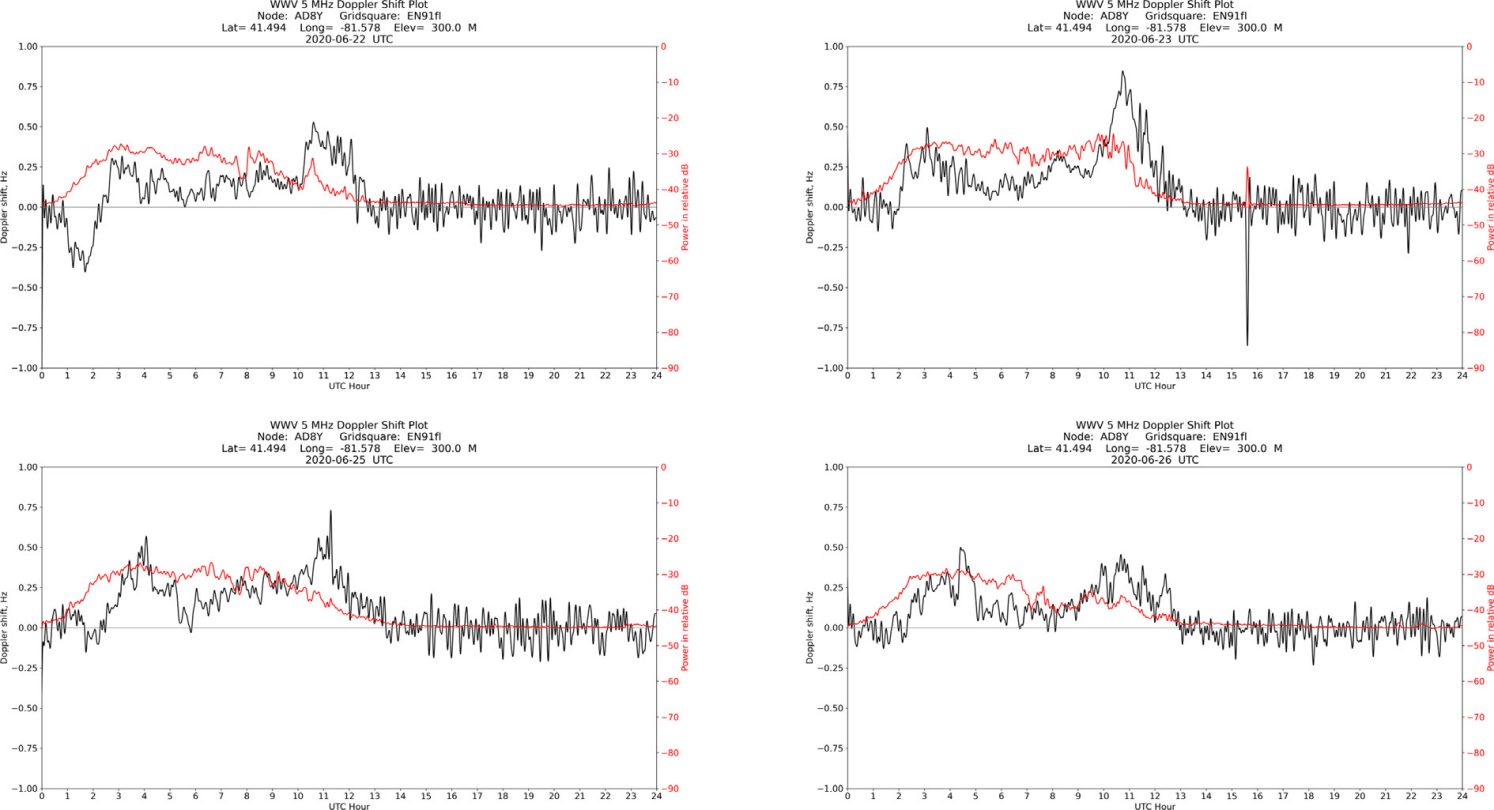
Plots from preceding (top row) and succeeding (bottom row) days for comparison to Fig. 10, showing the range of daily variation. Note that a sunrise peak is visible in each plot.

As indicated by the title block, the data in Fig. 10 was taken by station AD8Y in Cleveland, Ohio on June 24, 2020. The plot shows the frequency and amplitude of WWV’s carrier on 5 MHz over the course of a UTC day. An annotation has been added to indicate the sunrise peak. Plots from the same week are shown in Fig. 12: despite significant variation throughout the day, each plot shows a sunrise peak. Over time, the data shows solar cycle variations, seasonal variations, and movement of the sunrise with the changing length of day.

**Fig. 13:**
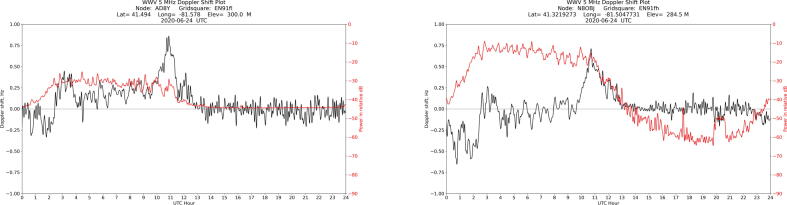
Plots from two nearby stations, made on the same day, show shared features.

The best verification method is to deploy two stations in the same local area and compare data. Fig. 13 shows plots made from WWV’s 5 MHz carrier from two stations about eight miles apart. Shared features in the frequency plots, like the distinct peaks between 03:00 and 05:00 UTC, are a strong indicator of geophysical variation.

Capabilities of the Grape Version 1:

• Low-cost monitoring of WWV and WWVH on 2.5, 5, and 10 MHz
• Daily plotting to facilitate scientific engagement.

## Declaration of Competing Interest

The authors declare that they have no known competing financial interests or personal relationships that could have appeared to influence the work reported in this paper.
